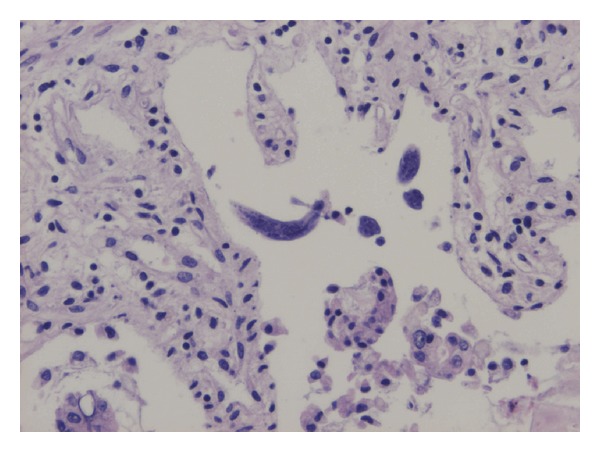# Fatal *Strongyloides* Hyperinfection Complicating a Gram-Negative Sepsis after Allogeneic Stem Cell Transplantation: A Case Report and Review of the Literature

**DOI:** 10.1155/2013/860976

**Published:** 2013-07-10

**Authors:** Isabel Izquierdo, Javier Briones, Rafael Lluch, Cristina Arqueros, Rodrigo Martino

**Affiliations:** ^1^Department of Hematology, Hospital Santa Creu i Sant Pau, C/Mar Casasnovas 90, 08041 Barcelona, Spain; ^2^Department of Hematology, Hospital Arnau de Vilanova, C/San Clemente 12, 46015 Valencia, Spain; ^3^Department of Oncology, Hospital Santa Creu I Sant Pau, C/Mar Casasnovas 90, 08041 Barcelona, Spain

## Abstract

*Strongyloides stercoralis* is an intestinal nematode that causes strongyloidiasis, which affects 30 to 100 million people worldwide. Risk factors for hyperinfection and disseminated disease include immunosuppressive drug therapy, human T-lymphotropic virus-1 (HTLV-1) infection, solid organ and bone marrow transplantation, hematologic malignant diseases, hypogammaglobulinemia, and severe malnutrition and associated conditions. The diagnosis can be difficult because a single stool examination fails to detect larvae in up to 70% of the cases, and the symptoms are nonspecific. Although eosinophilia is a common finding in patients with chronic *Strongyloides* infection, it is an unreliable predictor of hyperinfection. Furthermore, the lack of eosinophilia while receiving immunosuppressive therapy cannot reliably exclude the underlying chronic *Strongyloides* infection. We report here a fatal *Strongyloides* hyperinfection in a patient receiving allogeneic stem cell transplantation; risk factors and outcome in this clinical setting are discussed.

## 1. Introduction

*Strongyloides stercoralis* and *Strongyloides fuelleborni* are two intestinal nematodes that cause strongyloidiasis, which affects an estimate of 30 to 100 million people worldwide. The most common and globally distributed human pathogen of clinical importance is *S. stercoralis*.

A deregulation of the host's immune response during this latent infection may be lethal because multiple infective larvae can develop and invade other organs in addition to the intestine, causing sepsis and even death (hyperinfection syndrome) [1].

The clinical manifestations of *S. stercoralis* hyperinfection vary widely. Healthy individuals are usually asymptomatic even if chronically infected. The immunocompromised host is at risk for disseminated *Strongyloides* infection after transplant [2].

The diagnosis of strongyloidiasis should be suspected if there are clinical signs and symptoms (maculopapular rash, dyspnea, gastrointestinal symptom, etc.) or peripheral blood eosinophilia [3, 4]. A careful history including demographic data (residence in) or travelling to endemic areas should be assessed before transplantation [3]. 

Eosinophilia is usually the only indication of the presence of *S. stercoralis* infection [4]. However, although eosinophilia is a common finding in patients with chronic *Strongyloides* infection, it is an unreliable predictor of hyperinfection. Moreover, in chronic strongyloidiasis, eosinophilia may be present only intermittently [3–6], and, in fact, lack of eosinophilia while receiving immunosuppressant therapy cannot reliably exclude underlying chronic *Strongyloides* infection [1]. 

Laboratory diagnosis of strongyloidiasis is primarily based on the detection of *Strongyloides stercoralis* larvae by microscopic examination of stool samples [3–10]. 

Treatment of chronic intestinal strongyloidiasis should be performed before the immunosuppression therapy, if possible. Failure or relapse after antiparasitic therapy appears to be more frequent among immunosuppressed patients [3]. 

## 2. Case Report

A 36-year-old Bolivian male was admitted to our hospital with jaundice, increased liver transaminases, abdominal pain, and bloody diarrhea 62 days after allogeneic stem cell transplantation from his HLA-identical sibling. 

He was diagnosed with refractory anemia with excess blasts-1 and multilineage dysplasia in his country 5 years prior to the transplant. The patient was treated solely with monthly blood transfusions. 

On March 2011, he underwent allogeneic HSCT following a reduce-intensity conditioning with fludarabine 40 mg/m^2^ given orally for 5 days and busulfan 1 mg/kg given orally for 2 days. Graft-versus-host disease (GVHD) prophylaxis was done with cyclosporine A (CsA) 120 mg twice daily since day −8 and mycophenolate mofetil (MMF) 1275 mg every 8 h since day 0. 

Two months before transplantation, he was hospitalized for a pyoderma gangrenosum in his right elbow that was treated with prednisone (1 mg/kg) until the time of transplantation. 

The first month of the transplant was uneventful, except for a moderate renal failure on day +1, which required discontinuation of CsA and replaced by sirolimus.

Neutrophil and platelet engraftment occurred on day +18 and +23, respectively, and the patient was discharged in good condition 24 days after the transplantation. 

On day +64, the patient was hospitalized for crampy abdominal pain of a 4-5 day duration associated with bloody diarrhea and jaundice. He was taking prednisone (10 mg/day) for his pyoderma gangrenosum and MMF. (Sirolimus was discontinued on day +60 due to renal failure.)

 The patient had no fever, and his vital signs were normal. The physical examination was also normal. The patient presented painful abdominal palpation with neither hepato- nor splenomegaly. 

Laboratory findings on admission showed a hemoglobin concentration of 112 g/L, and white blood cell count of 1290/*μ*L with 320 neutrophils, 700 lymphocytes, and no eosinophils, and the platelet count was 32 × 10^9^/L. The biochemistry showed elevations of the total bilirubin: 131 *μ*g (normal ref. < 17), alkaline phosphatase: 1085 U/L (normal ref. 40–130), alanine aminotransferase: 802 U/L (normal ref. < 41), aspartate aminotransferase: 441 U/L (normal ref. < 37) and gamma-glutamyl-transpeptidase: 992 U/L (normal ref. < 54); creatinine was 206 *μ*mol/L (normal ref. < 106). An abdominal ultrasonography and computed tomography were performed to exclude an obstructive biliary disease. Hepatic GVHD was suspected, and prednisone (2 mg/kg) was initiated. A liver biopsy was performed (not suggestive of GVHD).

Six days after the biopsy, the patient developed respiratory failure, hypoxia (PH 7.19, pCO_2_ 36 mmHg, pO_2_ 32 mmHg), and severe abdominal pain. Hepatic and kidney failure were found in biochemistry. He was apyrexial. An abdominal CT was repeated and a subcapsular liver haematoma appeared. The chest X-ray showed minimal left-sided pleural effusion. Echocardiogram showed a 30% ejection fraction. At this stage, empirical therapy with meropenem, amikacine, ganciclovir, and liposomal amphotericin B was initiated.

 His general condition deteriorated rapidly developing hypotension and respiratory failure. The patient required intubation and mechanical ventilation. He developed progressive septic syndrome requiring high dose inotropics with no clinical response. 

The blood and urine cultures were positive for *Escherichia coli*. Stool examination showed numerous *Strongyloides stercoralis* filariform larvae. Septic shock by *E. coli* and *S. stercoralys* hyperinfections was considered the main diagnostics. Steroids and all immunosupressors were discontinued, and the patient was started on rectpl ivermectin and nasogastric albendazole.

Despite the ventilatory and hemodynamic supports, the patient's clinical condition gradually deteriorated, and the patient died 15 days after admission. 

Autopsy was performed, showing gastrointestinal bleeding with extensive and diffuse ulceration of small intestine, colon, and rectum with the presence of multiple *S. stercoralis* egg-filled fusiform larvae (Figure 1), bilateral pulmonary hemorrhage, acute pancreatitis, and tubular necrosis with nephrosclerosis.

## 3. Discussion

Strongyloidiasis is present in tropical and subtropical climates. It is endemic in sub-Saharan Africa, the West Indies, South America, Southeast Asia, Bangladesh, Pakistan, and the southeastern United States, as well as in eastern European countries such as Romania. An increased number of cases seen in developed countries are related to the increased numbers of immigrants, travelers, and refugees [3–6]. Low socioeconomic status, alcoholism, white race, and male gender have been associated with a high prevalence of *S. stercoralis* stool positivity. Some occupations such as farming or coal mining have been associated with the increased risk of infection, due to contact with soil contaminated with human waste. Generally, humans are infected transcutaneously. Drinking or swimming in contaminated water has not been proven to be a significant source of transmission.

The risk factors for hyperinfection and disseminated disease include immunosuppressive drug therapy (particularly steroids), human T-lymphotropic virus-1 (HTLV-1) infection, solid organ and bone marrow transplantation, hematologic malignant disease, especially lymphoma, hypogammaglobunemia, and severe malnutrition and associated conditions [5, 11].

The most common risk factor is steroids use. This results in a decrease of eosinophils that translates into an activation of the organism and increased egg production [7].

Coinfection with HTLV-1 seems to be a marker for poor prognosis in the patient developing hyperinfection, due to low serum IgE antibody levels; the patients who are diagnosed with HTLV-1 usually fail to antihelminthic therapy. 

Acute infection can result in localized maculopapular or urticarial rash at the site of larval entry [3, 5, 11]. Pulmonary symptoms include throat irritation, dry cough, dyspnea, and wheezing with peripheral eosinophilia [5, 6, 11]. Patients with hyperinfection who have peripheral eosinophilia appear to have a better prognosis [5, 11].

Gastrointestinal symptoms can develop several weeks later, just before the appearance of the larvae in the stool. 

Chronic strongyloidiasis is asymptomatic in more than 50% of patients infected with *Strongyloides*, and 75% of patients have fluctuating mild (5–15%) eosinophilia [3, 6]. Clinical symptoms reported by the patients are nonspecific such as nausea, diarrhea, constipation, anorexia, anal pruritus, and abdominal pain [3, 5–7, 11]. 

Hyperinfection syndrome develops when immunosuppression reduces the usual immune surveillance [3]. This is a phenomenon in which the larvae highly proliferate in the duodenum, migrate through the bowel to the venous system, and reach extraintestinal regions, especially in the lungs [3, 4, 6]. Massive hyperinfection in immunosuppressed patients can produce severe enterocolitis and widespread dissemination to heart, lung, and central nervous system [12]. This can produce worsening of pulmonary function with cough, wheezing, and hoarseness, and in some patients it can progress to an acute respiratory failure and death [3, 5].

Hyperinfection syndrome has a wide spectrum of gastrointestinal symptoms, including abdominal pain, diarrhea, nausea [3–5], or severe manifestations including paralytic ileus, obstruction and gastrointestinal bleeding [3, 6]. Other syndromes have, been described like *Strongyloides*-related glomerulonephritis and minimal change nephritic syndrome [11], liver abscess, pancreatitis, cholecystitis, or meningitis [3, 5]. Bacterial and fungal infections often occur in cases of hyperinfection because of the leakage of gut flora from bowel damage by moving larvae [4]. Bacteriemia due to Gram-negative enteric organisms (i.e., *Escherichia coli*, *Enterococcus faecalis*, and *Klebsiella pneumonia*) is a frequent complication [3]. The mortality of *Strongyloides* hyperinfection exceeds 80% [6, 9, 11].

Eosinophilia is usually the only indication to the presence of *S. stercoralis* infection [4]. However, although eosinophilia is a common finding in patients with chronic *Strongyloides* infection, it is an unreliable predictor of hyperinfection. Moreover, in chronic strongyloidiasis, eosinophilia may be present only intermittently [3–6], and, in fact, lack of eosinophilia while receiving immunosuppressant therapy cannot reliably exclude underlying chronic *Strongyloides* infection [1]. 

 Patients with risk factors for acquiring strongyloidiasis, that is, those who are being diagnosed with HTLV-1 or who undergo immunosuppressive drug therapy, should be screened [3–7, 11, 13]. Testing for strongyloidiasis should be done in all immunocompromised patients with epidemiological exposure and clinical symptoms [3, 5, 6]. There are no data regarding the cost effectiveness of screening for strongyloidiasis before transplantation [3]. 

Laboratory diagnosis of strongyloidiasis is primarily based on the detection of *Strongyloides stercoralis* larvae by microscopic examination of stool samples [3–10]. Techniques to detect rhabditiform larvae in stool include direct smear in saline-lugol iodine stain, formalin-ethyl acetate concentration, harada-Mori filter paper culture, and nutrient agar plate cultures [4, 6]. A single stool examination fails to detect larvae in up to 70% of cases [4, 7], while the diagnostic sensitivity for *S. stercoralis* increases to 60–70% with 3 or more stool samples [6, 8, 9] and can approach 100% if 7 consecutive daily stool specimens are examined [3, 4, 6, 7].

 Even when stool specimens are unreliable, an ELISA test for detecting the serum IgG against a crude extract of the larvae of *S. stercoralis* may be positive [6, 8–11]. 

*Strongyloides* antibody testing can be falsely negative in immunocompromissed patients [6, 14]. False-positive test can occur because of the presence of other helminth infections [3, 4, 13]. 

In *Strongyloides* hyperinfection, filariform larvae increase exponentially and can be detected in respiratory fluid, peritoneal fluid [3, 5, 6], blood, bone marrow, or cerebrospinal fluid [13]. 

 Biopsy of duodenum can also be used to document the presence of larva, and this is a frequent method of diagnosis for transplant recipients [3, 11]. 

All patients infected with *S. stercoralis* should be treated. No standard regimen exists for immunocompromised patients with hyperinfection or disseminated disease [7]. Antiparasitic therapies such as albendazole, thiabendazole, and ivermectin are the main used drugs. Ivermectin proved more effective than Albendazole in the treatment of strongyloidiasis that is limited to the gastrointestinal tract [15] and is considered the first-line therapy of *Strongyloides* hyperinfection. The recommended dose is 200 mg/kg (orally), given once daily for 2 days in intestinal forms and until larvae clearance for the treatment of disseminated strongyloidiasis or hyperinfection syndrome [7, 11]. If patients are unable to tolerate oral medication or if intestinal absorption is impaired, there are documented cases of successful treatment using veterinary intravenous formulations of ivermectin [16]. There are also case reports of response in patients treated with ivermectin enema [17]. The second choice consists in albendazole 400 mg or thiabendazole 25 mg/kg given orally twice a day for 3 days in chronic intestinal forms or until clearance of larvae for hyperinfection syndrome. Immunosuppressed patients have been reported to fail multiple courses of these commonly used agents; for this reason, combination therapy with ivermectin plus thiabendazole or albendazole in patients with disseminated disease has been recommended, although more experience and data are needed to determine the most effective treatment [11]. 

A cautious reduction in immunosuppression therapy is required [3]. In addition, patients should be evaluated and treated for concomitant bacterial infections.

Patients with *S. stercoralis* hyperinfection or dissemination are infectious, so contact isolation is recommended to prevent nosocomial transmission [4].

Hematopoietic stem cell transplant recipients have particularly poor outcomes with a mortality reported of up to 85% [6]. 

We have found seven cases reported in the literature of severe strongyloidiasis after hematopoietic stem cell transplantation. Five of them (71%) died due to complications of *Strongyloides* hyperinfection [1, 6, 9, 11, 12]. One patient initially cleared the infection but died later due to CMV infection [17], and one survived [8]. The two patients who survived were those who had an early diagnosis and the treatment was initiated (orally) before any organ failure. In all death patients, ivermectin was administrated intravenously, subcutaneously, or through the nasogastric tube, due to the critical conditions of the patients.

A relevant question in the allogeneic transplantation setting is if we should do a testing for strongyloidiasis in all immunocompromised patients with epidemiologic exposure or clinical symptoms before transplantation. There is only one systematic study in the literature of expanded pretransplant infectious screening in the Hispanic population [13]. They study a total of 83 patients, 5 tested positive for *S. stercoralis* (serology). All were treated with a single dose of ivermectine without complications. Based on these findings, Fitzpatrick et al. [2] suggest that screening of this population is warranted, although the sample size of their study is too small to make a sounding conclusion, and thus further studies are required to make a consensus on appropriate pretransplant screening. 

An important clinical issue is the use of a prophylaxis therapy for those patients with proven strongyloidiasis infection before allogeneic transplantation. Schaffel et al. have reported a double-blind randomized study comparing thiabendazole and placebo given once every month to all the patients from endemic areas. They do not find a benefit of giving prophylaxis, with similar incidences of strongyloidiasis and fewer side effects in the placebo group [10]. 

Although Spain is not an endemic country, our center receives patients coming from areas of high endemicity to undergo transplantation. Given the risk of strongyloidiasis in this population and the availability of an effective therapy that can save the life of the patient, we consider a pretransplant screening with ELISA test necessary in those patients, which has a positive predictive value of 91% and a negative predictive value of 98% [18]. Detection of *Strongyloides* IgG does not discriminate between current and previous infections so in patients with a positive test we should make a direct identification of *Strongyloides* larvae through the examination of at least 3 stool samples of the patient.

## Figures and Tables

**Figure 1:**